# Curriculum Reinforcement Learning Based on K-Fold Cross Validation

**DOI:** 10.3390/e24121787

**Published:** 2022-12-06

**Authors:** Zeyang Lin, Jun Lai, Xiliang Chen, Lei Cao, Jun Wang

**Affiliations:** Command & Control Engineering College, Army Engineering University of PLA, Nanjing 210007, China

**Keywords:** deep reinforcement learning, automatic curriculum learning, K-fold cross validation, replay buffer

## Abstract

With the continuous development of deep reinforcement learning in intelligent control, combining automatic curriculum learning and deep reinforcement learning can improve the training performance and efficiency of algorithms from easy to difficult. Most existing automatic curriculum learning algorithms perform curriculum ranking through expert experience and a single network, which has the problems of difficult curriculum task ranking and slow convergence speed. In this paper, we propose a curriculum reinforcement learning method based on K-Fold Cross Validation that can estimate the relativity score of task curriculum difficulty. Drawing lessons from the human concept of curriculum learning from easy to difficult, this method divides automatic curriculum learning into a curriculum difficulty assessment stage and a curriculum sorting stage. Through parallel training of the teacher model and cross-evaluation of task sample difficulty, the method can better sequence curriculum learning tasks. Finally, simulation comparison experiments were carried out in two types of multi-agent experimental environments. The experimental results show that the automatic curriculum learning method based on K-Fold cross-validation can improve the training speed of the MADDPG algorithm, and at the same time has a certain generality for multi-agent deep reinforcement learning algorithm based on the replay buffer mechanism.

## 1. Introduction

In recent years, deep reinforcement learning has developed rapidly in the field of artificial intelligence. By applying multilayer neural networks to approximate the value function of reinforcement learning, the perception ability of deep learning and the decision-making ability of reinforcement learning can be effectively combined, such as Atari games [1,2], complex robot motion control [3], and the application of AlphaGo intelligence in Go [4], etc.

Compared with single-agent reinforcement learning, multi-agent reinforcement learning algorithms can communicate, cooperate, and complete tasks together. Current mainstream multi-agent cooperative control algorithms include Lenient Deep Q Network (DQN) [5], Value-Decomposition Networks (VDN) [6], Q Mixing Network (QMIX) [7], Deep Recurrent Q-Network (DRQN) [8], Counterfactual Multi-Agent Policy (COMA) [9], Multi-Agent Deep Deterministic Policy Gradient (MADDPG) [10], Multi-Agent Proximal Policy Optimization (MAPPO) [11], etc. Compared to other algorithms, the Multi-Agent Deep Deterministic Policy Gradient (MADDPG) algorithm can also be applied to cooperative cooperation and competitive adversarial scenarios, and global observation and policy functions between agents can be used to accelerate the convergence of actor network and critic network in the training process [12], and can use the mechanism of centralized training and decentralized execution to enhance the stability and convergence of the algorithm, which has a broader application prospect in the current field of artificial intelligence field.

In applying reinforcement learning algorithms to real-world problem solving, the algorithms consume a lot of training time and training costs in interacting with the real environment (e.g., crash loss in training self-driving vehicles). To address this problem, we use curriculum learning in the reinforcement learning training process to facilitate the transfer of experience between samples.

Curriculum learning is distinguished by the ability to control the order of task selection and creation fully, thus optimizing the performance of the reinforcement learning target task [13]. When a reinforcement learning agent is faced with a difficult task, curriculum learning can assign auxiliary tasks to the agent to gradually guide its learning trajectory from easy to complex tasks until the target task is solved. At the 2009 International Conference on Machine Learning, Bengio et al. pointed out that the curriculum learning approach can be viewed as a special kind of continuous optimization approach that can start with smoother (i.e., simpler) optimization problems and gradually add coarser (i.e., more difficult), non-convex (norm) optimization problems to eventually perform the optimization of the target task [14].

Current mainstream multi-agent deep reinforcement learning algorithms can reduce the correlation between training samples and ensure independence between samples by applying the experience replay buffer mechanism in the model fitting process [15]. Automatic curriculum learning [14] can build a task sampler based on the experience replay buffer. According to the curriculum learning rule, extract the most suitable tasks for the current reinforcement learning agent from the experience replay buffer from easy to difficult for neural network training. In this way, the sum of the cumulative reward value of the agent is maximized, and the learning efficiency and robustness of deep reinforcement learning are improved.

To reasonably sort the learning order of task samples in automatic curriculum learning, this paper proposes an automatic curriculum learning method based on K-Fold Cross Validation, which can be combined with a deep reinforcement learning algorithm based on the experience replay buffer represented by MADDPG to improve the performance of reinforcement learning agents for task training.

This method defines the simple curriculum learning task as a task that the current reinforcement learning model can solve well, and divides automatic curriculum learning into the curriculum difficulty evaluation stage and the curriculum sorting stage. In the evaluation stage of curriculum difficulty, dividing the state training samples in the replay buffer space into K equal parts, multiple model networks are trained in parallel. With the rest of the teacher network model, the curriculum difficulty of the target state sample is evaluated, scored, and summed by a loss function and sum it up as the basis for the evaluation of the problem of task samples; In the stage of curriculum sorting, through the K-level classification of state samples from easy to difficult, the sample capacity in each difficulty category is randomly sampled for model learning. Finally, the training curriculums of the reinforcement learning algorithm are sorted from easy to difficult. Due to the applicability of the MADDPG algorithm in dealing with most multi-agent cooperative/competition problems, this paper uses the MADDPG algorithm as the basic reinforcement learning algorithm to combine the curriculum learning framework based on K-Fold cross validation and proposes a curriculum reinforcement learning algorithm based on K-Fold cross validation (KFCV-CL).

To test the effectiveness and feasibility of the KFCV-CL algorithm proposed in this paper, we applied it in two multi-agent reinforcement learning environments to verify its versatility and practicality. In the research process of the curriculum reinforcement learning method based on K-Fold cross validation, our research difficulties were the design of the curriculum ranking criteria and the design of the algorithm details of the sample ranking in the experience replay buffer, during which we compared the strengths and weaknesses of the existing algorithm research and proposed a new method of curriculum reinforcement learning based on the existing research. The experimental results show that the automatic curriculum learning method based on K-Fold cross validation can reflect more robust learning efficiency and robustness in combination with the MADDPG algorithm, which can shorten the training time and improve the learning performance.

The automatic curriculum learning mechanism based on K-Fold Cross Validation has the following characteristics:

(1) In sorting deep-reinforcement learning curriculums, this method can distinguish difficulty based on mutual measurement between target tasks, maintain the independence of task samples in the difficulty evaluation process, avoid interference with prior experience in curriculum sorting, and has better versatility for tasks.
(2) This method adopts the method of sampling from the replay buffer space in the process of automatic curriculum learning, which can be applied to the current mainstream multi-agent deep reinforcement learning algorithm (based on the replay buffer space mechanism), and has good algorithm applicability.

The rest of this paper is as follows. Section 2 introduces related work, and Section 3 presents the MADDPG algorithm and the basic concepts of automatic curriculum learning. Section 4 introduces the automatic curriculum learning method based on K-Fold Cross Validation in detail. Section 5 compares and analyzes experimental results and discusses them accordingly, and Section 6 presents the conclusions.

## 2. Related Work

The concept of an easy-to-difficult training method can be traced back to the curriculum learning method proposed by a scientific research team led by Bengio, a leader in machine learning, at the International Conference on Machine Learning (ICML) in 2009 [14]. In the learning process, the curriculum can be regarded as a sequence of the training process. The teacher model divides the entire machine learning task into several subparts. Bengio et al. have proven the advantages of curriculum policy in image classification and language modeling through experiments. This inspired us to design reinforcement learning tasks using curriculum learning algorithms.

In the early stages of the application of curriculum learning to the reinforcement learning process, most people adopt the method of human-designed curriculum for curriculum sorting instead of automatically generating curriculum by agents, including learning to execute short programs [16] and finding the shortest paths in graphs [17]. By experimenting with applied methods of curriculum learning, Felipe et al. [18] proposed that curriculums can be automatically generated from object-oriented task descriptions, using the generated curriculums to reuse knowledge across tasks, Jiayu Chen et al. [19] perform curriculum updates and agent number expansion in the process of automatic curriculum learning, Daphna et al. [20] proposed the Inference Curriculum (IC) method, a way of transferring knowledge from another network, trained on different tasks.

Regarding the design method of curriculum reinforcement learning, our method is similar to the safe curriculum reinforcement learning method proposed by Matteo Turchetta et al. [21], the teacher-student curriculum learning presented by Tambet Matiisen et al. [22], and the curriculum reinforcement learning policy learning proposed by Sanmit Narvekar et al. [23] is relatively close. Our method divides reinforcement learning curriculum sorting into two stages, curriculum difficulty assessment and curriculum sorting by stages. Matteo Turchetta et al. avoided the dangerous policy of student network by resetting the controller, Tambet Matiisen et al. set teacher-student curriculum learning method, the teacher model selects the task with the highest learning slope from the given task set for students to learn, Sanmit Narvekar et al. defined the curriculum sorting problem as a Markov Decision Problem (MDP). The above methods are pretty different from our proposed KFCV-CL method by extending the model to handle reinforcement learning problems.

Our method can optimize curriculum learning in two aspects: model assessment and model selection. Traditional curriculum learning mainly conducts model assessment through autonomous assessment and heuristic assessment, and the heuristic assessment method requires direct curriculum sample assessment based on experts’ empirical knowledge, which suffers from intense subjectivity and weak generalization ability. In contrast, the model autonomous assessment method conducts a direct curriculum sample. The autonomous model evaluation method directly assesses the curriculum sample through student models, which suffer from inefficient data utilization and overfitting problems. Our approach uses time difference error as the curriculum evaluation criterion and the K-fold cross validation method, which can avoid the overfitting problem caused by the autonomous assessment model, thus obtaining a more reasonable and accurate assessment of the student model and improving the accuracy of curriculum selection in curriculum learning.

## 3. Background

In this section, we introduce the basic concepts of the MADDPG algorithm, the PER-MADDPG algorithm, and automatic curriculum learning.

### 3.1. Multi-Agent Deep Deterministic Policy Gradient Algorithm Model

#### 3.1.1. Basic Knowledge of Deep Reinforcement Learning

**Figure 1:**
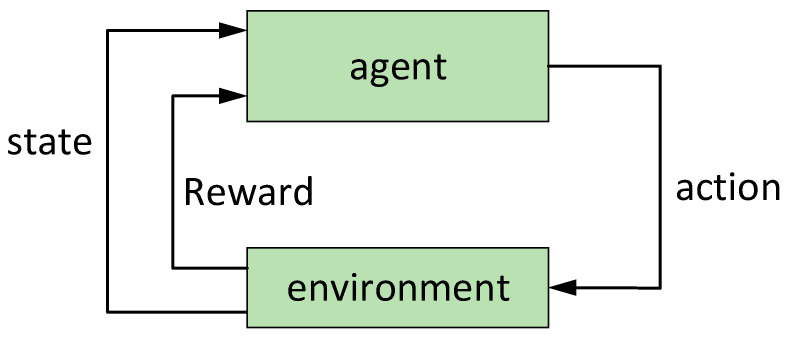
Schematic diagram of reinforcement learning.

Deep reinforcement learning uses the value function approximation of deep neural networks to solve reinforcement learning problems, deep reinforcement learning agents conduct a series of actions in a given environmental state to maximize the cumulative reward value. Such issues are often referred to as tasks and are formalized as the Markov Decision Problem [24] (MDP). Multi-agent deep reinforcement learning can be described in the form of a nine-tuple Markov decision, $M\left(n,\phi \right)=\langle n,\phi ,S,A,O,P,R,g_{\phi },\gamma \rangle$, where the discrete variable n represents the number of agents, the parameter ϕ∈Φ represents the initial state and target, S represents the state space, A represents the common action space of each agent, $P(s^{\prime }|s,a)$ represents the state transition probability of taking action a to state $s^{\prime }$ in the current state s, $P(s^{\prime }|s,a)$ represents the reward function obtained under the given state s and joint action $A=\left(a_{1},\ldots ,a_{n}\right)$, and γ represents the discount factor. The schematic diagram of reinforcement learning is shown in Figure 1.

Taking homogeneous agents as the basis for reinforcement learning training, each agent i learns a policy $\pi_{\theta }(a_{i}|o_{i},g)$ under the condition of a common goal with parameter θ. To measure the strengths and weaknesses of each agent’s policy $\pi_{\theta }(a_{i}|o_{i},g)$, state value function $V\left(n,\phi ,\pi_{\theta }\right)$ is introduced in multi-agent deep reinforcement learning, $V\left(n,\phi ,\pi_{\theta }\right)$ represents the cumulative expected reward value that n agents can obtain under the current state and target ϕ according to the policy $\pi_{\theta }$, Bellman Equation is as follows:

(1) $$V\left(s,\pi \right)=\sum_{a\in A}\pi \left(s,a\right)\left[R\left(s,a\right)+\gamma \sum_{s^{\prime }\in S}P\left(s,a,s^{\prime }\right)V\left(s^{\prime },\pi \right)\right]$$

Among them, γ represents the discount factor, S represents the state space, a represents the action space, R(s,a) represents the reward value a received after taking an action in the state s, representing the state transfer probability $P\left(s,a,s^{\prime }\right)$ of taking an action a in the current state s to transfer to the state s′, and the optimal policy $\pi^{*}$ refers to the policy that can maximize the cumulative reward value and its corresponding optimal value function $V^{*}\left(s\right)$ is expressed as follows:

(2) $$V^{*}\left(s,\pi \right)=\underset{a\in A}{max}\left\{R\left(s,a\right)+\gamma \sum_{s^{\prime }\in S}P\left(s,a,s^{\prime }\right)V^{*}\left(s^{\prime },\pi \right)\right\}$$

#### 3.1.2. PER-MADDPG Algorithm

In the cooperative/competitive environment of multi-agents, the policy π(a|s) of agent i will change with the policy of its competitors or partners, which is the problem of non-stationarity in the environment [25]. Since the MADDPG algorithm can better use global observation and policy functions to accelerate the training process and has better stability and convergence, this paper adopts the MADDPG algorithm as the basic algorithm of reinforcement learning.

**Figure 2:**
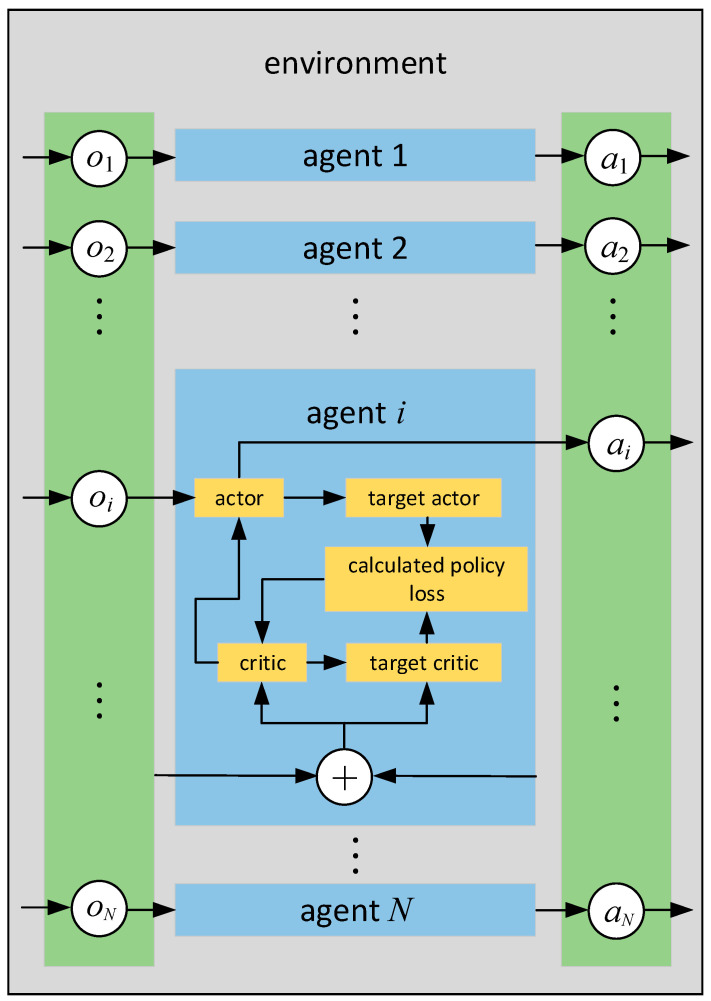
Diagram of MADDPG algorithm training framework.

MADDPG is an improved algorithm based on the Actor-Critic framework. Its algorithm training framework is shown in Figure 2. Assuming that there are N agents in the environment, the joint policy space is $\pi =\left(\pi \left(\theta_{1}\right),\pi \left(\theta_{2}\right),\ldots ,\pi \left(\theta_{N}\right)\right)$, and each agent adopts the single-agent DDPG policy framework. The structure of the training environment algorithm consists of Actor network, Critic network, Target Actor network, and Target Critic network, $\theta =\left(\theta_{1},\theta_{2},\ldots ,\theta_{N}\right)$ represents a collection of individual agent policy functions. In the figure, agent i is taken as an example, and other agents are represented by boxes.

The MADDPG algorithm agent adopts the principle of centralized learning and distributed application. Each agent i obtains the actions $a_{i}^{j}=\pi_{i}^{j}\left(o_{i}^{j}\right)$ corresponding to the current state s according to the current policy π, and stores the state experience samples obtained by interacting with the environment into the experience replay buffer space. For the MADDPG algorithm, the experience replay mechanism can reduce the correlation between training samples and ensure the independence between samples [26]. The experience replay space is usually implemented as a kind of circular buffer, which can be used to save the recently collected state sample quaternion $x_{t}\left(s_{t},a_{t},r_{t},s_{t+1}\right)$, indicating that the reinforcement learning agent takes action $a_{t}$ in the state $s_{t}$ during the exploration of the environment, obtains reward value and transfers to the state $s_{t+1}$ at the same time.

However, in the process of using state samples, the method of randomly extracting samples for learning ignores the difference of samples’ learning effects on agents. Through the application of Prioritized Experience Replay (PER) [15], the importance of each state sample can be sorted and used as a basis for the probability that the state sample will be drawn to ensure that the state sample is selected. The relationship between the probability P(i) and the state sample priority $p_{i}$ is monotonically increasing, and the sample selection probability of the lowest priority state is always greater than 0, that is

(3) $$P\left(i\right)=\frac{p_{i}^{a}}{\sum p_{k}^{a}}$$

(4) $$p_{i}=\frac{1}{rank\left(i\right)}$$

In the equation, $p_{i}>0$ represents the priority of state samples, a represents the degree of use of the priority of state samples, and rank(i) means the order of the state samples after the storage of the replay buffer storage is sorted according to the size of absolute value |δ| of temporal difference error of samples.

**Figure 3:**
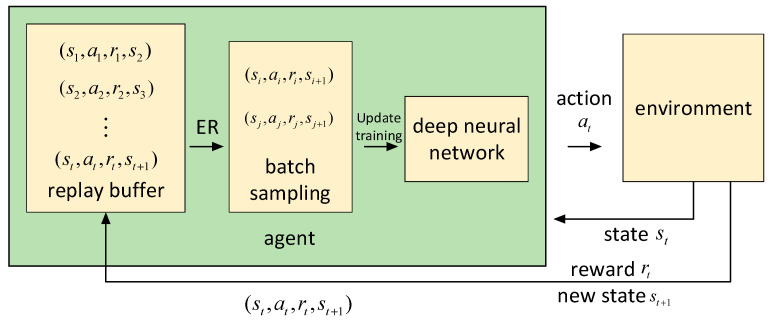
Schematic diagram of experience replay buffer.

After all agents complete the interaction with the environment, each agent extracts the experience from the experience replay buffer space according to the order of priority experience replay and uses it for training its neural network. The schematic diagram of the experience replay buffer is shown in Figure 3.

To enhance the learning effect of the agent learning process, the input of the critic network includes the observations and action spaces of other agents. $Q=Q\left(s^{m},a_{1},a_{2},\ldots ,a_{N},\theta^{Q}\right)$, where $s^{m}=\left(o_{1}^{m},o_{2}^{m},\ldots ,o_{N}^{m}\right)$, the parameters of the Critic network model are updated by minimizing the temporal difference error loss function, where the calculation equation of the loss function is as follows:

(5) $$L=\frac{1}{M}\sum_{m=1}^{M}{\left(y_{m}-Q\left(s^{m},a_{1},a_{2},\ldots ,a_{N},\theta^{Q}\right)\right)}^{2}\;$$

where the y function represents the cumulative average future reward of agent i in the target actor network, and the loss function L is the square of the difference between the *y* function and the *Q* value of the original network. The Actor network parameters are updated by the stochastic gradient descent method. The gradient descent equation is as follows:

(6) $$\nabla_{\theta^{\pi }}J=\frac{1}{M}\sum_{m=1}^{M}\nabla_{\theta^{\pi }}\pi \left(o,\theta^{\pi }\right)\nabla_{a}Q\left(s^{m},a_{1},a_{2},\ldots ,a_{N},\theta^{Q}\right)$$

where o and $a_{N}$ represents the observed value and action of the ith agent, $\pi \left(o,\theta^{\pi }\right)$ represents that the agent selects the action following the policy network, $\nabla_{\theta^{\pi }}J$ represents that the optimal Q value is selected and gradient descent is performed in the direction of the action π, K represents the number of empirical samples drawn each time i.e., the size of batchsize, and $\nabla_{\theta^{\pi }}\pi \left(o,\theta^{\pi }\right)$ represents that the action of agent i is selected according to the policy π.

### 3.2. Automatic Curriculum Learning

In most reinforcement learning, the training effect of the agent depends on the difficulty of training task samples and the total training time. However, existing reinforcement learning algorithms still have difficulties and challenges in applying them to many scenarios, such as high sampling complexity and weak convergence [27]. In addition, reinforcement learning agents can only conduct model training to improve their performance after adequate information interaction with the environment. However, due to the problems of reward sparsity, partial observability, delay, high-dimensional observation space, and action space, it is prone to the problem that the reinforcement learning algorithm cannot converge.

The main idea of curriculum learning is to construct a task sampler q(n,ϕ) based on experience replay buffer. The task sampler can extract the most suitable tasks M(n,ϕ) for the current agent training in real-time to maximize the cumulative reward value J(θ) of training.

For a given number of agents n, simplify J(θ) to maximize the expected value $J={Ε}_{\phi ∼p\left(\phi \right)}\left[V\left(\phi ,\pi \right)\right]$, where p(ϕ) represents the uniform distribution of ϕ over the range of possible values. Then the lower bound of the value of J can be obtained as follows:

(7) $$\begin{array}{ll} J & =E_{\phi ∼p}\left[V\left(\phi ,\pi \right)\right]=E_{\phi ∼q}\left[\frac{p\left(\phi \right)}{q\left(\phi \right)}V\left(\phi ,\pi \right)\right]\\ & =E_{\phi ∼q}\left[V\left(\phi ,\pi \right)+\left(\frac{p\left(\phi \right)}{q\left(\phi \right)}-1\right)V\left(\phi ,\pi \right)\right]\\ & \geq \underset{J_{1}:policy\;update}{\underbrace{E_{\phi ∼q}\left[V\left(\phi ,\pi \right)\right]}}+\underset{J_{2}:curriculum\;update}{\underbrace{E_{\phi ∼q}\left[V\left(\phi ,\pi \right)\log\frac{p\left(\phi \right)}{q\left(\phi \right)}\right]}} \end{array}$$

where, for all ϕ, the equality sign of the inequality holds if and only if p(ϕ)=q(ϕ).

The cumulative reward value J(π,q) is decomposed into policy update reward $J_{1}$ and curriculum update reward $J_{2}$, where $J_{1}$ represents the policy update target under the premise of the current task curriculum q(ϕ) and $J_{2}$ represents the curriculum update target that can be achieved by changing q(ϕ). Therefore, it can be proved that the cumulative reward value J(π,q) of reinforcement learning can be maximized by updating the policy π to maximize $J_{1}$ and by updating the curriculum q(ϕ) to maximize $J_{2}$, thereby generating a curriculum learning framework.

In the curriculum learning algorithm, the reinforcement learning agent extracts state samples from the replay storage space for learning, and this process is similar to the process of learning students according to the prescribed curriculum plan. Curriculum learning generates state task samples by adjusting the elements of the MDP process corresponding to the continuous task space. The process can be described as follows: the agent selects relatively simple state samples from the replay storage in the initial stage of algorithm learning for reinforcement learning and then continuously increases the training difficulty of the task curriculum and conducts curriculum learning as the basis of the final training target task. However, for a reinforcement learning agent in a locally observable state, the artificial pre-setting of the curriculum sequence may not be applicable, the simple training samples selected in the initial state may be difficult to train, and the selected difficult samples can be easily grasped by the agent. Therefore, the learning sequence of the curriculum tasks can be determined by reinforcement learning agents, and the current state samples that are most suitable for agents to learn from the experience replay buffer can be independently selected, which is called automatic curriculum learning [28].

There is a crucial problem in deep reinforcement learning algorithms based on automatic curriculum learning: how to design an explicit evaluation criterion to evaluate and sort the complexity of task samples in replay storage space [29]. In the traditional automatic curriculum learning method, the difficulty of the curriculums is sorted by the absolute value of temporal difference error, and the samples whose loss value exceeds the threshold δ are considered difficult samples. This method is general, but the loss value is directly used as the sample difficulty index. It introduces uncontrollable distribution errors, which may lead to overfitting of the state samples themselves and non-convergence of the algorithm.

After the difficulty of given state samples is sorted in the curriculum task sorting stage, previous work usually manually controls the use of samples according to the sample difficulty or directly discards easy and difficult samples to conduct curriculum training. Discarding easy and difficult samples may lead to a loss of information and affect the fit of deep learning models. How to implement training programs on the premise of losing as little information as possible is a significant problem facing current automatic curriculum learning.

## 4. Curriculum Reinforcement Learning Algorithm Based on K-Fold Cross Validation

This paper proposes a general curriculum learning framework, the K-Fold Cross Validation Curriculum Learning (KFCV-CL) framework, which can be applied to multi-agent deep reinforcement learning problems based on the replay buffer mechanism. By analyzing the temporal difference loss values of all training state samples in the cooperative/competitive task, we found that the reinforcement learning agent can quickly grasp the state task (simple task) whose initial state is closer to the final state after a few rounds of training, but it is difficult to grasp the state task (a difficult task) whose initial state is far from the final state during the whole training process. Based on this correlation, this work proposes a K-Fold Cross Validation method to define the state task difficulty. The K-Fold Cross Validation curriculum learning framework is divided into two stages: curriculum difficulty evaluation and curriculum sorting.

For all target tasks, let D be the training set of all state samples used for training and θ be the set of parameters of the neural network model used for training. In the curriculum difficulty evaluation stage, a curriculum difficulty score is given to each state sample in D, and C is used as the difficulty level score set corresponding to all state samples in the training set D. In the curriculum sorting stage, based on the above difficulty level scores, D is divided into a series of task curriculum sets $\left\{S_{i}:i=1,2,\ldots ,K\right\}$ from easy to difficult for training, and finally, the expected complex task training effect is obtained.

### 4.1. Assessment of Curriculum Difficulty

The difficulty of a curriculum task sample can be determined by many elements, for example, the distance between the initial state of each agent and the final state, the number, and location of obstacles in the environment, the sparsity of rewards, etc. The difficulty of curriculum tasks can be distinguished explicitly by setting a difficulty evaluation function. However, traditional difficulty assessment methods have the following problems. First, the weight coefficients between the difficulty factors are not easy to assign. For example, it is difficult to distinguish the influence between the degree of reward sparsity and the number of obstacles. Second, traditional methods are not universal for multi-agent reinforcement learning problems in different environments, and the influence of factors needs to be reevaluated.

The curriculum difficulty evaluation method proposed in this paper can evaluate the relative difficulty of the target sample through the teacher network formed by the remaining samples, avoid the subjective bias caused by the assignment of the weight coefficient, and improve the versatility of the difficulty evaluation method.

The MADDPG algorithm belongs to the Temporal Difference (TD) algorithm, which uses the estimated reward function $R_{t+1}+\gamma V\left(S_{t+1}\right)$ to update the value function $V\left(S_{t}\right)$ (TD(0))

(8) $$V\left(S_{t}\right)\leftarrow V\left(S_{t}\right)+a\left(R_{t+1}+\gamma V\left(S_{t+1}\right)-V\left(S_{t}\right)\right)$$

where $R_{t+1}+\gamma V\left(S_{t+1}\right)$ is called TD target and $R_{t+1}+\gamma V\left(S_{t+1}\right)-V\left(S_{t}\right)$ is called TD error.

We take the absolute value of temporal difference error as the evaluation reference for the difficulty of the curriculum task because the state sample with a significant total value of temporal difference error may harm the fitting of the training model. There are the following reasons: 1. In a complex environment, the reward value is easily affected by random noise, which may affect the real network training and lead to deviations in target network training; 2. In the stochastic gradient descent process of deep neural networks, state samples with larger absolute values of temporal difference errors require smaller update steps to reach the global minimum of gradient descent.

To evaluate the difficulty of the curriculum, the state sample training set D in experience replay buffer space in the reinforcement learning algorithm is equally divided into K parts, $\left\{{\tilde{D}}_{i}:i=1,2,\ldots ,K\right\}$, and the K training sample subsets are used for parallel training of the reinforcement learning model respectively, $\left\{{\tilde{\theta }}_{i}:i=1,2,\ldots ,K\right\}$, and the resulting K network models are called teacher network models, which are used to evaluate the samples in the target student network model. The training process of the teacher network model is described as follows:

(9) $${\tilde{\theta }}_{i}=\underset{{\tilde{\theta }}_{i}}{argmin}\sum_{d_{j}\in {\tilde{D}}_{i}}L\left(d_{j},{\tilde{\theta }}_{i}\right)\;i=1,2,\ldots ,K$$

where L represents the loss function of temporal difference error, $d_{j}\in \tilde{D}$ represents the experience samples drawn from the training set of state samples in the experience replay buffer D, and $\left\{{\tilde{\theta }}_{i}:i=1,2,\cdots ,K\right\}$ represents the K reinforcement learning models trained by a subset of K training samples, respectively.

After the teacher network training is completed, the difficulty evaluation of curriculum task samples is carried out. The task sample $d_{j}$ is included in the kth subset, the teacher network corresponding to the kth subset is removed, and the remaining K−1 teacher models are used to estimate the score of the curriculum task sample $d_{j}$, and the K−1 scores are obtained. The scoring process of each teacher model for the target curriculum task sample $d_{j}$ is expressed as follows:

(10) $$c_{ji}={\left(y-Q_{teacher}^{\pi }\left(s,a_{1},a_{2},\ldots ,a_{N}\right)\right)}^{2}\;y=r_{j}+\gamma Q^{\pi^{\prime }}\left(s^{\prime },a_{1}^{\prime },a_{2}^{\prime },\ldots ,a_{N}^{\prime }\right){|}_{a_{i}^{\prime }=\pi_{i}^{\prime }\left(o_{i}\right)}$$

where $c_{ji}$ represents the score of the target curriculum task sample $d_{j}$ by the teacher network corresponding to the ith subset, $Q_{\mathrm{teacher}}\left(s_{j},a_{j}\right)$ represents the Q value obtained by inputting the state $s_{j}$ and action value $a_{j}$ into the teacher model, $a_{N}$ represents the action taken by the Nth agent, and γ represents the discount factor, then the difficulty score corresponding to the task sample $d_{j}$ is defined as the sum of the scores of all remaining teacher training models:

(11) $$c_{j}=\sum_{i\in \left(1,\ldots ,K\right),i\neq k}c_{ji}$$

**Figure 4:**
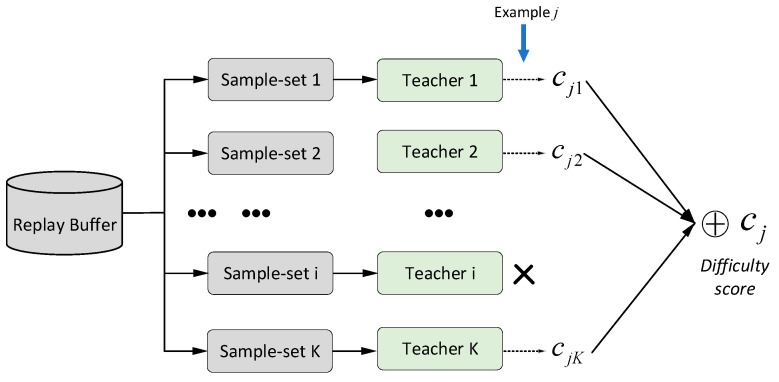
Framework diagram to assess the difficulty of curriculum tasks.

As a result, the curriculum difficulty score corresponding to the task sample $d_{j}$ can be obtained. Since teacher training models are carried out independently in the scoring process, the influence of the data contained in the task sample $d_{j}$ on the task sample, the difficulty score can be avoided, the robustness and accuracy of the scoring result can be improved, and it has good versatility. Figure 4 shows the framework for assessing the difficulty of curriculum tasks.

### 4.2. Curriculum Sorting Rules

In this section, we divide the training samples in the experience buffer space D into multiple stages for reinforcement learning training according to the difficulty scores corresponding to the previously calculated curriculum task samples. $\left\{S_{i}:i=1,2,\ldots ,K\right\}$, at each stage of training, the random sampling method is still used for model training for this part of the state samples to ensure the independent and identical distribution of the samples and avoid overfitting.

After estimating the difficulty of all training task samples according to the task curriculum difficulty measurement method in the previous section, the training samples are sorted in difficulty order from low to high, the task samples are divided into K training sets, $C_{1}-C_{K}$, and the training sets are sorted from easy to difficult.

For task state samples, this hierarchical structure of difficulty scores can directly reflect the inherent difficulty distribution corresponding to the training sample set. After that, we divide the learning of the curriculum into K stages. For each learning stage, random sampling is conducted from the training set of the corresponding difficulty for periodic training of the neural network. After the training period $S_{K}$ is reached, the final stage of training is carried out through the set D of the original training samples. The training set is used to ensure the convergence of the model to finally realize the curriculum reinforcement learning process from easy to difficult.

(12) $$\frac{1}{K}num\left(C_{1}\right):\frac{1}{K}num\left(C_{2}\right):\cdots :\frac{1}{K}num\left(C_{K}\right)$$

### 4.3. Algorithm Framework and Process

The pseudocode of the KFCV-CL algorithm is as follows (Algorithm 1):
**Algorithm 1: KFCV-CL algorithm****for** episode=1 to max−episode **do**  Initialize a random process N for reinforcement learning action exploration  **for** t=1 to max−episode−length **do**    for each agent v, select the action $a_{v}=\pi_{v}\left(o_{v},\theta^{\pi_{v}}\right)+\varepsilon_{a}$ w.r.t the current policy     The agent v conducts action $a_{v}$, transfers to the next state $s_{v}^{\prime }$ and obtains the reward $r_{v}$    Store $\left(s,a,s^{\prime },r\right)$ into buffer space D    $s\leftarrow s^{\prime }$
    Divide the experience samples in D into K equal parts, $\left\{{\tilde{D}}_{i}:i=1,2,\ldots ,K\right\}$
Teacher models are trained for $D_{i}$, the parameters are $\left\{{\tilde{\theta }}_{i}:i=1,2,\ldots ,K\right\}$ ${\tilde{\theta }}_{i}=\underset{{\tilde{\theta }}_{i}}{argmin}\underset{d_{j}\in {\tilde{D}}_{i}}{\sum }L\left(d_{j},{\tilde{\theta }}_{i}\right)$
The score $c_{j}=\underset{i\in \left(1,\ldots ,K\right),i\neq k}{\sum }c_{ji}$ of each experience sample $d_{j}$ is obtained $c_{ji}={\left(y-Q_{teacher}^{\pi }\left(s,a_{1},a_{2},\ldots ,a_{N}\right)\right)}^{2}$ $y=r_{j}+\gamma Q^{\pi^{\prime }}\left(s^{\prime },a_{1}^{\prime },a_{2}^{\prime },\ldots ,a_{N}^{\prime }\right){|}_{a_{v}^{\prime }=\pi_{v}^{\prime }\left(o_{v}\right)}$
    Sort the experience samples and divide them into K training sets $C_{1}-C_{K}$
    **for** agent v=1 to N **do**      Sampling a minibatch of experience quadruples $\left(s^{m},a^{m},s^{\prime m},r^{m}\right)$ from D
      Calculate the expected action reward of each experience sample            $y_{m}=r_{v}^{m}+\gamma Q^{\prime }\left(s^{\prime m},a_{1}^{\prime },a_{2}^{\prime },\ldots ,a_{N}^{\prime },\theta^{Q^{\prime }}\right)$      Minimize loss function to update Critic network parameters          $L=\frac{1}{M}\overset{M}{\underset{m=1}{\sum }}{\left(y_{m}-Q\left(s^{m},a_{1},a_{2},\ldots ,a_{N},\theta^{Q}\right)\right)}^{2}$      Update the Actor network parameters by the following gradient function          $\nabla_{\theta^{\pi }}J=\frac{1}{M}\overset{M}{\underset{m=1}{\sum }}\nabla_{\theta^{\pi }}\pi \left(o,\theta^{\pi }\right)\nabla_{a}Q\left(s^{m},a_{1},a_{2},\ldots ,a_{N},\theta^{Q}\right)$
    **end for**    Update the target network parameters of each agent v         $\theta_{v}^{\prime }=\tau \theta_{v}+\left(1-\tau \right)\theta_{v}^{\prime }$
  **end for****end for**

In summary, we can learn curriculum tasks from easy to difficult through the difficulty assessment and curriculum sorting process. Next, we will conduct simulation experiments in two cooperative/competitive task environments.

## 5. Experiment

We conduct simulation validation of the curriculum reinforcement learning algorithm based on K-Fold cross validation in two experimental environments in multi-agent particle world environment [30]. Each group of experiments is carried out in Ubuntu18.04.3+PyTorch+OpenAI environment, the hardware environment is IntelCore i7-9700K+GeForceRTX2080+64G memory, the test environment tasks are the cooperative task (Hard Spread), and adversarial task (Grassland), the birth positions of agents are randomly generated, and obstacles are randomly added, which can better enhance the difficulty combined with the algorithm simulation needs in this paper.

The reinforcement learning testing process does not have an input dataset but relies on data interaction with a simulated environment. The learning performance of the reinforcement learning algorithm can be measured from two aspects: 1. The cumulative reward value changes with the number of training episodes. Under the premise of the same number of training episodes, the higher the total reward value obtained by agents, the better the training effect, and the faster the convergence speed; 2. From the actual performance of the agents in the simulation environment, the agents can cooperate and compete in the environment and have better performance, indicating that the training efficiency of the algorithm is higher. The key hyperparameters set for the RL training process are listed in Table 1.

### 5.1. Hard Spread Experiment

**Figure 5:**
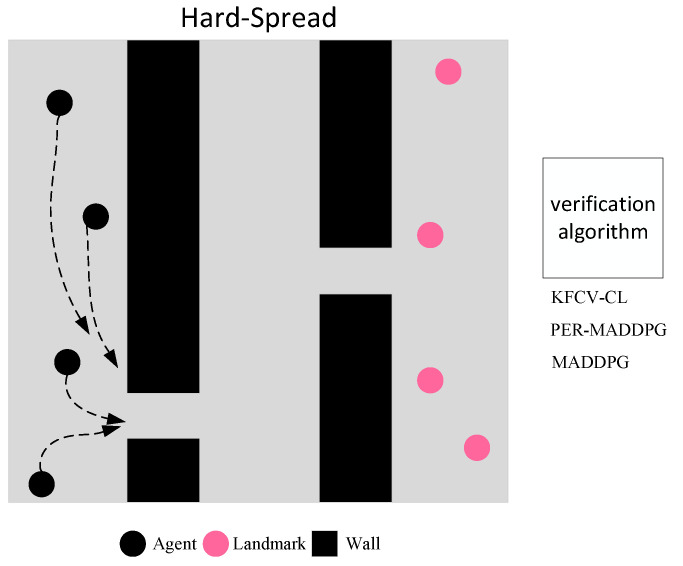
Schematic diagram of training in a cooperative environment.

The multi-agent cooperation experiment adopts the “Hard Spread” environment. As shown in Figure 5, in a square two-dimensional plane with a side length of 6, the plane is isolated into three parts by randomly adding walls, and N agents and N landmarks are generated randomly in the plane. The agents can only observe the landmark position but cannot observe the wall. The learning goal of agents is to reach a landmark position in the shortest time and to avoid collision with other agents or walls. To achieve the overall training goal as soon as possible, each agent needs to consider the distance to the nearest landmark and, at the same time, consider the relative positions of other agents and the target to obtain the globally optimal policy and avoid falling into the trap of local minimum.

The reward obtained by each agent at each time step consists of three parts, including 1. the distance between the agent and the nearest landmark; 2. whether the agent collides with other agents or walls; 3. whether the agent has reached a landmark location. The closer the distance between the agents and the landmarks, the higher the reward, and the environment gives a positive reward to the agent that covers the landmark and a negative reward to the agent that collides. The agent collision reward is defined as follows:

(13) $$C=\{\begin{array}{ll} -1, & if\;collided\\ 0, & if\;not\;collided \end{array}$$

The agent coverage landmark reward is defined as follows:

(14) $$D=\{\begin{array}{ll} +4, & if\;covered\\ 0, & if\;not\;coverd \end{array}$$

Let the distance between agent i and landmark j be K(i,j), $K\left(i,j\right)=\sqrt{{\left(x_{i}-x_{j}\right)}^{2}+{\left(y_{i}-y_{j}\right)}^{2}}$, represents the closer the agent i is to the nearest target j, the greater the reward value it gets. Then the reward function of agent i at each moment is

(15) $$r_{i}=-\phi \left[\underset{j\leq N}{min}\left(K\left(i,j\right)\right)\right]+C+D$$

In the value of the equation, ϕ represents the eigenvector corresponding to the shortest distance.

As shown in Figure 5, in the N=4 environment, the KFCV-CL, PER-MADDPG, and MADDPG algorithms are used to control the movement of the agent. Under the same number of training episodes, the algorithm that obtains a higher cumulative reward value and the cover rate has higher training performance and faster model convergence.

**Figure 6:**
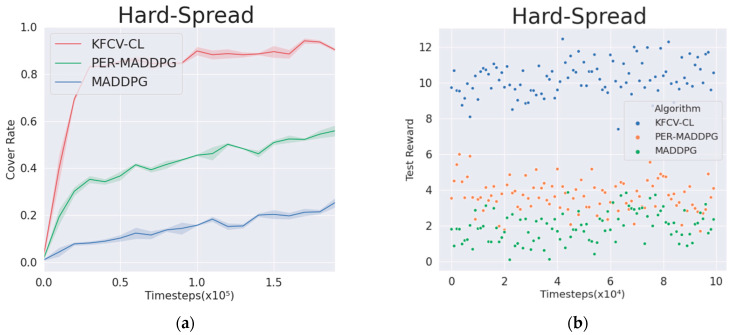
Representation diagram of agents in the cooperative environment. (**a**) Target cover rate in the cooperative environment. (**b**) Test reward in the cooperative environment.

Figure 6a shows the average cover rate curves of the three algorithms after $2\times 10^{5}$ rounds of training. It can be seen from the figure that the coverage of the KFCV-CL algorithm can be significantly improved compared with the PER-MADDPG and MADDPG algorithms after sorting the curriculums from easy to difficult.

Table 1 shows the average number of collisions in each step, the average minimum distance between the agent and the nearest landmark, and the average number of landmarks covered by the agent after $1.9\times 10^{5}$ rounds of training and 10,000 random rounds of experiments based on the policies learned by the agents. From the results in Table 2, the agents controlled by the KFCV-CL algorithm can significantly improve the three indicators. As shown in the figure, the test reward obtained by the agent performing random 10,000 rounds of experiments can be stably maintained at a high level, which means that the agents can learn a better policy. Figure 6b is a scatter diagram of the test reward obtained by the agents performing random 10,000 rounds of experiments.

### 5.2. Grassland Experiment

In a cooperative training environment, the agent’s policy will continue to mature along with the training process, and the cumulative reward value will continue to increase. In a multi-agent adversarial environment, the agent’s policy will also be affected by the opponent’s agent policy, and the convergence speed of the two policies is not synchronized, so the cumulative reward value will fluctuate and will have higher requirements for algorithm training performance, the KFCV-CL algorithm can effectively improve the learning performance of agents in the mutual game.

The multi-agent adversarial experiment adopts the “grassland” experiment, which is adapted from the classic experiment “predator-prey” in the MPE environment and improves the confrontation and training difficulty of the experimental environment by introducing grass and allowing the agent (sheep) to die. In the two-dimensional plane [0, 1], there are $N_{S}$ sheep, $N_{W}$ wolves and L piles of grass, where the sheep move twice as fast as the wolf, and the grass stays in place.

All environments in the grassland experiment are entirely observable. The agent needs to adjust its policy according to the opponent’s policy and the partner’s policy and respond to the global state. As the number of wolves increases, it becomes increasingly difficult for sheep to survive. At the same time, the sheep become more brilliant, which will also reversely motivate the wolves to improve their policies. The reward value of the wolf i depends on the distance between it and the sheep and whether the sheep are caught. The smaller the distance between each other, the greater the reward value.

Let the distance between the wolf i and the sheep j be K(i,j), then

(16) $$K\left(i,j\right)=\sqrt{{\left(x_{i}-x_{j}\right)}^{2}+{\left(y_{i}-y_{j}\right)}^{2}}$$

When a wolf collides with (eats) a sheep, the wolf will receive a higher reward value for it. At the same time, the sheep will receive a negative reward value (penalty). Reward value for successfully colliding (eating) the sheep of the wolf is as follows:

(17) $$C_{1}=\{\begin{array}{ll} +5, & if\;captured\\ 0, & if\;not\;captured \end{array}$$

To maintain the excellent operation of the training environment and prevent the agent from giving up learning a better policy because of escaping the boundary, a large negative reward value (penalty) is imposed on the agent that escapes the boundary of the two-dimensional plane. The size of the penalty depends on how far the agent leaves the boundary, and the boundary reward is

(18) $$C_{2}=\{\begin{array}{ll} 0, & \max(x_{i},y_{i})<0.9\\ -200\left(\max(x_{i},y_{i}\right)-0.9), & \max(x_{i},y_{i})\geq 0.9 \end{array}$$

When the sheep encounters (eats) grass, it will get a reward value. After the grass is eaten by sheep, it will respawn in another random area.

(19) $$C_{3}=\{\begin{array}{ll} +2, & if\;captured\\ 0, & if\;not\;captured \end{array}$$

In the grassland experiment, wolves cooperate to complete the predation task. Since the overall reward value is prioritized in the environment, the instantaneous higher reward of the individual agent can be sacrificed, so the distance taken when defining the reward value is the minimum distance between the wolves and sheep, and the reward function of the wolf i is

(20) $$r_{i}=-\phi \left[\underset{i\leq n}{min}K\left(i,j\right)\right]+C_{1}+C_{2}$$

The movement speed of the sheep group is faster than that of the wolf group, and the movement range has boundaries. Therefore, to maximize the overall reward value of the sheep group, considering the sum of the distances from the wolves in the environment, the reward function of sheep j is:

(21) $$r_{j}=\phi \left[\sum_{i=1}^{N}\left(K\left(i,j\right)\right)\right]-C_{1}+C_{2}+C_{3}$$

In the above equation, ϕ represents the eigenvector corresponding to the shortest distance.

**Figure 7:**
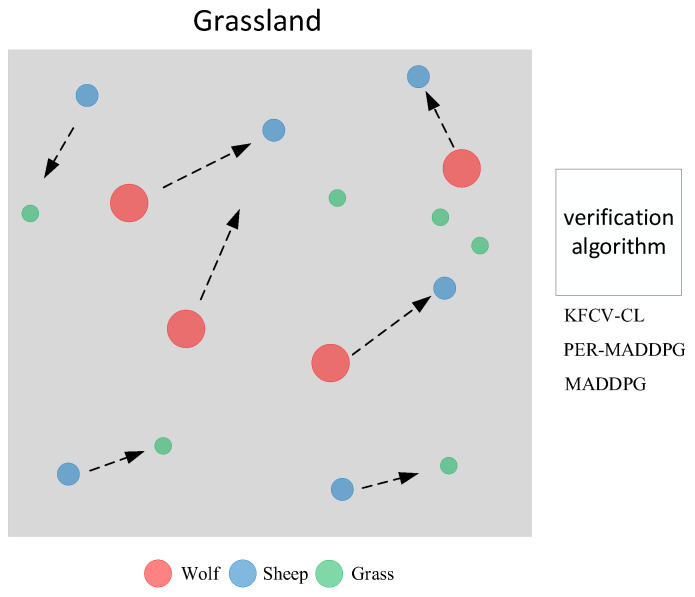
Schematic diagram of training adversarial environment. Red balls represent the wolf agents, the blue balls represent the sheep, and the green balls represent the grass. The wolf agent and the sheep agent are controlled by the algorithms, respectively.

In the reinforcement learning environment, the KFCV-CL, PER-MADDPG, and MADDPG algorithms are used to control wolves to move and compete with sheep trained by other baseline algorithms. The average reward value for wolves is used as the wolf evaluation index, and the average reward value for sheep is used as the sheep evaluation index. Figure 7 shows a schematic diagram of the training in the adversarial environment training.

**Figure 8:**
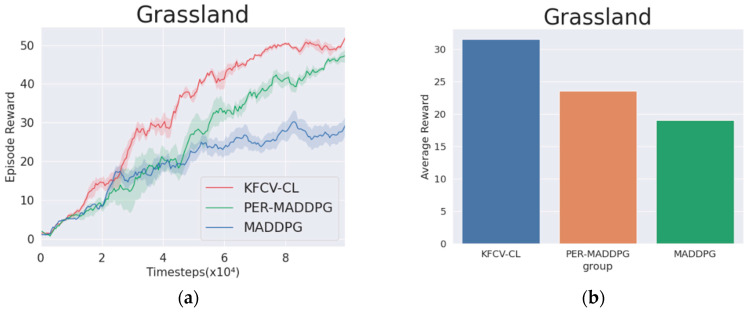
Representation diagram of agents in an adversarial environment. (**a**) The episode reward is achieved in the adversarial environment. (**b**) The average reward obtained by the three algorithms in the adversarial environment.

In the environment of $N_{S}=12,\;N_{w}=16$, the KFCV-CL, PER-MADDPG, and MADDPG algorithms are used to control the wolves to move and compete with the sheep trained by MADDPG. The curves in Figure 8a represent the trend of the reward value obtained by the wolf agent corresponding to the three algorithms in $1\times 10^{5}$ rounds, the legend of (b) illustrates the histogram of the average reward value obtained by the three algorithms in 10,000 rounds. From the reward value curves of the adversarial experiment, it can be concluded that due to asynchrony in the algorithm training process, the reward value curve has a fluctuating upward process. The red curve corresponding to the KFCV-CL algorithm is significantly higher than the reward curves corresponding to the other two algorithms. The average reward value of KFCV-CL is 1.34 times that of PER-MADDPG and 1.65 times that of MADDPG, which shows that the performance of the KFCV-CL algorithm is significantly better than the other two algorithms.

**Figure 9:**
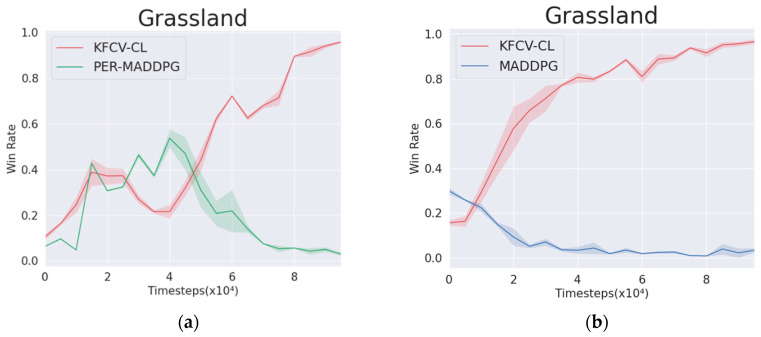
The win rate of wolf and sheep using two algorithms, respectively, in the adversarial environment. (**a**) KFCV-CL versus PER-MADDPG. (**b**) KFCV-CL versus MADDPG.

As shown in Figure 9, in the environment of $N_{S}=12,\;N_{w}=16$, the KFCV-CL algorithm is used to control the wolf agent, and the PER-MADDPG and MADDPG algorithms are used to control the sheep agent, respectively, and the winning rate curves corresponding to the wolf and sheep are obtained. From the winning rate curves, the wolf agent controlled by the KFCV-CL can gain an advantage in a short time (20,000 rounds) against the sheep agent controlled by the MADDPG algorithm, and the winning rate is stable above 0.85. However, when the wolf agent controlled by KFCV-CL fights against the sheep agent controlled by PER-MADDPG, it wins and loses in the early stage but can learn better policies in the later stage, thereby gaining an advantage in the confrontation. It can be seen that the performance of the KFCV-CL algorithm is significantly better than the other two algorithms.

## 6. Conclusions

To solve the problems of complex curriculum sorting and slow convergence in curriculum reinforcement learning, this paper proposes a curriculum reinforcement learning method based on K-Fold Cross Validation, which can automatically sort curriculums through curriculum difficulty assessment and curriculum sorting without relying on expert experience, and it can be applied in multi-agent deep reinforcement learning algorithm based on replay buffer space. Through simulations in the cooperative environment and the adversarial environment, the usability and superiority of the algorithm was proven, which is of solid research and practical significance. The reinforcement learning method based on automatic curriculum learning has the advantages of fast solution speed and strong model generalization ability for solving optimization problems in industrial practice, and can provide new approaches and new ideas for solving combinatorial optimization problems. Our method has the advantages of high data utilization and accurate curriculum evaluation and can avoid the overfitting problem in the evaluation process, but there are still problems such as the long training period and insufficient applicability for particular scenarios of reinforcement learning (such as sparse reward environment and multi-agent body confidence distribution problem). T he subsequent study will be conducted on how to combine automatic curriculum learning with algorithms of value decomposition network structure and further adaptation to multi-agent body. In the future, we will further investigate how to combine automatic curriculum learning with algorithms of value decomposition network structure, how to further adapt to a multi-agent simulation environment, and how to reduce the time complexity of curriculum sequencing further.

## Figures and Tables

**Table 1:** Parameter setting of the DRL process.

Parameters	Values
Discount factor	0.99
Size of RNN hidden layers	64
Size of the replay buffer	5000
Exploration	0.1
Initial curriculum factor λ	0.1
Batch size of the replay buffer	128
Learning rate of actor network	0.001
Learning rate of critic network	0.001
The update rate of the target network	0.01

**Table 2:** Data analysis table of 10,000 random experiments conducted by agents in the cooperative environment.

The Experimental Algorithm	The Average Number of Collisions	Mean the Closest Distance	The Average Number of Landmarks Covered
KFCV-CL	0.687	2.406	1.791
PER-MADDPG	0.707	2.529	1.288
MADDPG	0.742	3.636	0.701

## Data Availability

The data used to support the findings of this study are available from the website https://github.com/openai/multiagent-particle-envs (accessed on 26 July 2022).
